# Experimental *Actinobacillus pleuropneumoniae* challenge in swine: Comparison of computed tomographic and radiographic findings during disease

**DOI:** 10.1186/1746-6148-8-47

**Published:** 2012-04-30

**Authors:** Carsten Brauer, Isabel Hennig-Pauka, Doris Hoeltig, Falk FR Buettner, Martin Beyerbach, Hagen Gasse, Gerald-F Gerlach, Karl-H Waldmann

**Affiliations:** 1 Clinic for Swine and Small Ruminants, Forensic Medicine and Ambulatory Services, University of Veterinary Medicine Hannover, Bischofsholer Damm 15, D-30173, Hannover, Germany; 2 Institute for Cellular Chemistry, Hannover Medical School, Carl-Neuberg Strasse 1, D-30625, Hannover, Germany; 3 Department of Biometry, Epidemiology, and Information Processing, University of Veterinary Medicine Hannover, Bünteweg 2, D-30559, Hannover, Germany; 4 Institute of Anatomy, University of Veterinary Medicine Hannover, Bischofsholer Damm 15, D-30173, Hannover, Germany; 5 Institute of Microbiology, University of Veterinary Medicine Hannover, Bischofsholer Damm 15, D-30173, Hannover, Germany

## Abstract

**Background:**

In pigs, diseases of the respiratory tract like pleuropneumonia due to *Actinobacillus pleuropneumoniae (App)* infection have led to high economic losses for decades. Further research on disease pathogenesis, pathogen-host-interactions and new prophylactic and therapeutic approaches are needed. In most studies, a large number of experimental animals are required to assess lung alterations at different stages of the disease. In order to reduce the required number of animals but nevertheless gather information on the nature and extent of lung alterations in living pigs, a computed tomographic scoring system for quantifying gross pathological findings was developed. In this study, five healthy pigs served as control animals while 24 pigs were infected with *App*, the causative agent of pleuropneumonia in pigs, in an established model for respiratory tract disease.

**Results:**

Computed tomographic (CT) findings during the course of *App* challenge were verified by radiological imaging, clinical, serological, gross pathology and histological examinations. Findings from clinical examinations and both CT and radiological imaging, were recorded on day 7 and day 21 after challenge. Clinical signs after experimental *App* challenge were indicative of acute to chronic disease. Lung CT findings of infected pigs comprised ground-glass opacities and consolidation. On day 7 and 21 the clinical scores significantly correlated with the scores of both imaging techniques. At day 21, significant correlations were found between clinical scores, CT scores and lung lesion scores. In 19 out of 22 challenged pigs the determined disease grades (not affected, slightly affected, moderately affected, severely affected) from CT and gross pathological examination were in accordance. Disease classification by radiography and gross pathology agreed in 11 out of 24 pigs.

**Conclusions:**

High-resolution, high-contrast CT examination with no overlapping of organs is superior to radiography in the assessment of pneumonic lung lesions after *App* challenge. The new CT scoring system allows for quantification of gross pathological lung alterations in living pigs. However, computed tomographic findings are not informative of the etiology of respiratory disease.

## Background

For decades, respiratory tract diseases in pigs have led to high economic losses in pig producing countries. Acute disease outbreaks can often be immediately diagnosed based on the detection of clinical signs by farmers and veterinarians, while chronic and subclinical respiratory disorders are difficult to diagnose. In cases with chronic disease, gross pathology at abattoir slaughter provides the only indication of disease presence. In contrast to many diagnostic methods available for detecting bacterial and viral respiratory pathogens in samples of living pigs, methods to diagnose the extent of lung lesions in chronically diseased animals, usually showing no clinical signs, are very limited [1].

*Actinobacillus pleuropneumoniae* (*App*), the causative agent of pleuropneumonia in pigs is one of the most important bacterial lung pathogens in swine and is commonly used as a standard model for respiratory disease [2-5] and was therefore chosen for this study. *App* is considered both, as a primary and secondary pathogen. In combination with other viral and bacterial pathogens it belongs to the “Porcine Respiratory Disease Complex“(PRDC), representing a typical health problem in pigs [2,6]. Co-infection with *Mycoplasma hyopneumoniae* can lead to respiratory diseases with high morbidity and mortality [3,7-11].

During the peracute form of disease due to *App*, sudden death is often the sole finding without premonitory signs, while acute disease is characterised by fever, lethargy, cyanosis, frothy nasal discharge, dyspnea and coughing. Acute signs dissipate during the chronic stage of the disease which is associated with intermittent coughing, reduced growth and feed conversion efficiency [2,5]. Often, subclinically and chronically infected animals do not show any clinical symptoms. As *App* is commonly endemic in pig populations, antibody detection by serological tests does not enable discrimination between subclinical infection and disease [12-15].

Typical lung alterations during the acute phase of an *App* infection are necrotising and fibrohaemorrhagic pneumonia associated with pleurisy [16-18]. Fibrous adhesions between the parietal and visceral pleura and consolidations in the form of abscess-like nodular lesions encapsulated by connective tissue are a characteristic gross pathological finding in the chronic stage of the disease [5]. A tentative diagnosis of *App* presence can be made on the basis of the clinical signs [5], and gross pathology is considered characteristic but not pathognomonic [17]. Thus, only organism detection from affected lung tissue by bacteriology or PCR provides a definitive diagnosis [5,19,20].

New prophylactical and therapeutic approaches for respiratory diseases in pigs are crucial. Standardised animal challenge models including sequential examinations after experimental challenge are the current method of choice for clinical studies to evaluate such new approaches. However, these animal experiments require a large number of test animals, because necropsy has to be carried out successively at different time points.

The aim of the present study was to detect gross pathological lung lesions in living animals by computed tomography (CT). The diagnostic potential and limitation of this imaging method was analysed in comparison to digital radiography. Sequential euthanasia and necropsy of test animals during the course of this study were replaced by CT examinations. A CT scoring system for disease classification was developed, allowing for quantification of pneumonic lung alterations. CT findings during the course of the *App* infection were compared to findings from clinical, serological and radiological examinations. At the end of the study on day 21, CT findings were also compared to findings from gross pathology and histological examinations. Ultimately, the CT methodology is expected to reduce the number of test animals in scientific studies since sequential euthanasia becomes dispensable.

## Results

### Clinical examination

After challenge, the animals showed variable disease severity. On day 7 the clinical score ranged from 0.40 to 26.90 and on day 21 from 0.60 to 2.98 (Table 1). There were five animals on day 7 and three animals on day 21 free of clinical signs. Mild symptoms were observed in three animals on day 7 and in 17 animals on day 21. Two animals showed typical symptoms of *App* infection with a rise in body temperature up to 41.8°C, apathy, dyspnoea and anorexia on both examination days. Two animals (Nos 10, 9) were euthanized on days 1 and 3 post-challenge, respectively, due to generalised severe disease signs, including severe dyspnea. Nevertheless, they underwent CT and radiographic examination prior to gross pathological examination. Findings from those two animals were included in the day 7 analyses. No signs of respiratory disease were detected in the control group.

**Table 1:** List of individual respiratory disease scores in the control and challenge group

		**CT score**		**radiographic score**		clinical score*		**lung lesion score**	***App *reisolation**
	**pig**	**day 7**	**day 21**	**day 7**	**day 21**	**day 7**	**day 21**	**day 21**	**day 21**
control group	1	0.00	0.00	0.00	0.00	0.30	0.50	0.00	−
	2	0.00	0.21	2.00	1.00	0.72	0.10	7.04	−
	3	0.00	0.00	0.00	0.00	0.50	0.20	3.57	−
	4	0.00	0.00	1.00	1.00	0.60	0.70	0.00	−
	5	1.30	0.29	3.00	3.00	0.53	0.30	3.57	−
	median	0.00	0.00	1.00	1.00	0.53	0.30	3.57	
	mean	0.26	0.10	1.20	1.00	0.53	0.36	2.84	
	SD	0.58	0.14	1.30	1.22	0.15	0.24	2.95	
challenge group	6	0.00	0.00	1.00	2.00	0.40	1.30	0.00	−
	7	0.00	0.00	3.00	2.00	0.90	1.00	0.00	−
	8	1.10	1.10	16.00	16.00	3.07	0.81	6.32	+
	9^**1**^	5.20		36.00		19.10		31.43^**1**^	+^**1**^
	10^**2**^	6.92		32.00		26.90		20.00^**2**^	+^**2**^
	11	2.70	1.60	12.00	14.00	2.60	2.06	4.39	+
	12	2.97	2.21	15.00	12.00	2.80	1.03	6.35	+
	13	3.21	2.23	21.00	25.00	2.80	0.89	12.33	+
	14	0.00	0.00	2.00	2.00	0.60	0.60	0.00	−
	15	0.00	0.23	4.00	1.00	1.33	0.70	0.00	−
	16	1.34	1.07	12.00	11.00	2.26	0.73	5.40	+
	17	1.35	0.24	5.00	10.00	1.12	0.93	1.84	+
	18	2.05	3.71	26.00	23.00	1.96	2.98	21.65	+
	19	2.38	1.21	24.00	22.00	1.36	2.81	3.42	+
	20	2.45	1.39	13.00	10.00	1.89	0.70	3.00	+
	21	2.80	1.98	14.00	4.00	1.63	1.03	5.13	+
	22	0.00	0.00	10.00	12.00	0.60	1.90	0.00	−
	23	0.88	1.48	4.00	7.00	1.70	2.27	2.63	+
	24	1.23	0.00	4.00	8.00	0.50	1.80	0.00	+
	25	1.34	0.61	9.00	8.00	0.90	2.10	1.60	+
	26	2.15	1.79	26.00	15.00	2.57	1.87	8.46	+
	27	3.34	1.39	7.00	7.00	2.07	1.90	5.20	+
	28	0.30	0.00	10.00	15.00	0.70	1.60	0.00	+
	29	0.00	0.00	10.00	9.00	0.90	1.70	0.00	−
	mean	1.82	1.01	13.17	10.68	3.36	1.49	5.80	
	SD	1.75	1.00	9.73	6.81	6.21	0.70	8.05	

*Quantification of clinical signs by scoring system of Höltig et al. (2008). Day 7: not affected 0–0.7, slightly affected 0.71–7.13, moderately affected 7.14–13.56, severely affected >13.56. Day 21: not affected 0–2, slightly affected 2.01–34.70, moderately affected 34.71–67.3, severely affected >67.3.

^**1**^ day 3 post challenge (euthanized on day 3 post challenge), ^**2**^ day 1 post challenge (euthanized on day 1 post challenge), SD standard deviation.

### Radiographic examination

**Figure 1:**
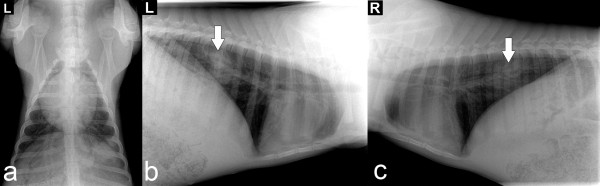
**Digital radiography of the thorax on day 21 post challenge. (a)** dorso-ventral view, **(b)** lateral view, left side,** (c)** lateral view, right side Arrows show focal lesions.

Radiologic images from diseased animals revealed diffuse shadings, increased tissue density around bronchi and shading overlapping the cardiac and diaphragmatic silhouettes (Table 2, Figure 1a, 1b and 1c).

**Table 2:** Evaluation of digital radiographic findings

**Finding**	**Points**
Heart shadow/visible diaphragmatic shadow	0
No increased tissue density around bronchi	0
No shading of the lung tissue	0
Heart shadow/diaphragmatic shadow visible, but superimposed by diffuse opacities	1
Mild increase in tissue density around bronchi	1
Mild diffuse shading of the lung tissue	1
Heart shadow/diaphragmatic shadow not visible	2
Severe increase in tissue density around bronchi	2
Severe diffuse shading of the lung tissue	2
Well-defined areas of high density	3

In the challenge group, radiographic scores ranged from 1 to 36 on both examination days (Table 1). Based on radiographic scores, 6 out of 29 pigs in the challenge group at day 7 and 5 out of 27 pigs at day 21 were classified as not diseased (radiographic score ≤ 4), while all 5 control pigs at days 7 and 21 were classified as not diseased (Table 1). Table 3a, 3b and 3d compare the allocation of animals to disease class by radiographic score at days 7 and 21 with their allocation to disease class by other diagnostic tests.

**Table 3:** Classification of disease severity using radiographic, lung lesion and CT scores

					
**a)** Classification of pigs based on disease severity assessed by CT score and radiographic score on day 7 post challenge					
CT score on day 7 post challenge	radiographic score* on day 7 post challenge				
	not affected 0–4	slightly affected 5–15	moderately affected 16–26	severely affected > 26	Total
severely affected (≥2.23)	0	5 (17. 2%)	2 (6.9%)	2 (6.9%)	9 (31.0%)
moderately affected (1.71–2.22)	0	0	2 (6.9%)	0	2 (6.9%)
slightly affected (0.24–1.70)	3 (10.3%)	4 (13.8%)	1 (3.5%)	0	8 (27.6%)
not affected (0–0.23)	8 (27.6%)	2 (6. 9%)	0	0	10 (34.5%)
Total	11 (37.9%)	11 (37.9%)	5 (17.3%)	2 (6.9%)	29 (100.00%)
**b)** Classification of pigs based on disease severity assessed by CT score and radiographic score on day 21 post challenge					
CT score on day 21 post challenge	radiographic score* on day 21 post challenge				
	not affected 0–4	slightly affected 5–15	moderately affected 16–26	severely affected > 26	Total
severely affected (≥ 2.23)	0	0	2 (7.4%)	0	2 (7.4%)
moderately affected (1.71–2.22)	1 (3.7%)	2 (7.4%)	0	0	3 (11.1%)
slightly affected (0.24–1.70)	1 (3.7%)	7 (26.0%)	2 (7.4%)	0	10 (37.1%)
not affected (0–0.23)	8 (29.6%)	4 (14.8%)	0	0	12 (44.4%)
Total	10 (37.0%)	13 (48.2%)	4 (14.8%)	0	27 (100.00%)
**c)** Classification of pigs based on disease severity assessed by CT score and lung lesion score					
CT score on day 21 post challenge	lung lesion score** on day 21 post challenge				
	not affected 0	slightly affected 0.1–5.0	moderately affected 5.1–10.0	severely affected > 10	Total
severely affected (≥ 2.23)	0	0	0	4 (13.8%)	4 (13.8%)
moderately affected (1.71–2.22)	0	0	3 (10.4%)	0	3 (10.4%)
slightly affected (0.24–1.70)	0	7^b^ (24.1%)	3 (10.3%)	0	10 (34.4%)
not affected (0–0.23)	10^a^ (34.5%)	1^b^ (3.5%)	1^b^ (3.4%)	0	12 (41.4%)
Total	10 (34.5%)	8 (27.6%)	7 (24.1%)	4 (13.8%)	29 (100.0%)
**d)** Classification of pigs based on disease severity assessed by radiographic score and lung lesion score					
radiographic score* on day 21 post challenge	lung lesion score** on day 21 post challenge				
	not affected 0	slightly affected 0.1–5.0	moderately affected 5.1–10.0	severely affected > 10	Total
severely affected>26	0	0	0	2 (6.9%)	2 (6.9%)
moderately affected16–26	0	1 (3.5%)	1 (3.5%)	2 (6.9%)	4 (13.8%)
slightly affected5–15	4 (13.8%)	5^b^ (17.2%)	4 (13.8%)	0	13 (44.8%)
not affected0–4	6^a^ (20.7%)	2^b^ (6.9%)	2^b^ (6.9%)	0	10 (34.5%)
Total	10 (34.5%)	8 (27.6%)	7 (24.1%)	4 (13.8%)	29 (100.00%)

*[[21]],**[[22]].

^a^including two control pigs, ^b^including one control pig.

### CT investigation

CT scores developed in this study ranged from 0 to 6.92 on day 7 and from 0 to 3.71 on day 21 in the challenge group (Table 1). Following the CT examination on day 7, six animals in the challenge group showed no pathological changes, while the other challenged animals showed ground-glass opacities or consolidations of varying degrees. At day 7, one of the 5 control pigs was classified as slightly affected (CT score 1.30).

**Figure 2:**
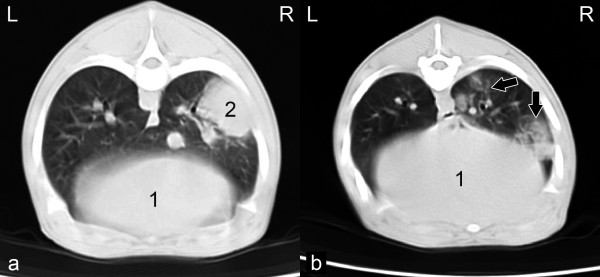
**CT-sectional image. (a)** of the thorax of a pig at the level of the 7th thoracic vertebra on day 21 post-challenge, sternal recumbent position, lung window (W:1500HU/L:-600HU), (1) liver, (2) hyperdense, focal consolidation in the right diaphragmatic lung lobe; **(b)** of the thorax of a pig at the level of the 8th thoracic vertebra on day 21 post-challenge, sternal recumbent position, lung window (W:1500HU/L:-600HU), (1) liver. Arrows show ground-glass opacities.

On day 21, consolidations and ground glass opacities were observed in 14 animals in the challenge group and also in 2 pigs in the control group (Table 1, Figure 2a, 2b). On days 7 and 21, 19% and 13% of the pathological changes were in the area of the apical lobes, respectively, 38% and 42% in the area of the cardiac lobe and 43% and 45% in the area of the diaphragmatic lobes, respectively.

In general, ground-glass opacities were recorded by transverse sections as hazy, diffuse and non-homogeneous areas of increased lung tissue density due to decreased aeration and characterised by distinct outlines of the bronchial tree and vasculature. In contrast, consolidations were focal, homogeneous areas, with defined margins and an increase in lung density. The underlying blood vessels and airway walls were obscured.The average density values (HU) of the consolidations were in the positive range of the Hounsfield Scale on both examination days after challenge.

### Gross pathology and bacteriology

Lung lesion scores ranged from 0 to 31.43. The highest lung lesion score in an animal surviving until day 21 was 21.65. At necropsy, eight challenged animals and two animals from the control group lacked any gross pathological changes (Table 1). Six animals from the challenge group and 2 animals from the control group were classified as slightly affected (Table 3 c, d). One animal in the control group classified as moderately affected had lung tissue consolidation within the right apical lobe that was considered atypical of *App* infection, on the basis of histological and bacteriological findings (cartarrhal and purulent bronchopneumonia with isolation of *Bordetella bronchiseptica* and *Mycoplasma flocculare*) and negative for *App*. A slight interstitial pneumonia was examined in all control animals by histological examination.

**Figure 3:**
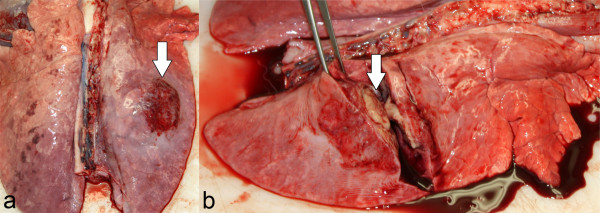
**Lung changes caused by *App *(a and b) on day 21 post challenge.** Arrows show abscess-like nodules encapsulated in connective tissue.

29% of gross lung lesions were located in the apical, 24% in the cardiac and 47% in the diaphragmatic lobes. The cut surfaces of the multiple, dark red and demarcated consolidated foci were coloured grey-red to off-white (Figure 3a and 3b). In addition, in 16 pigs from the challenge group, fibrinous pleurisy and adhesions were detected. Many pigs showed abscess-like nodules encapsulated in connective tissue. One of the two pigs euthanized before day 7 showed excessive serosanguinous fluid in the thoracic cavity. In both pigs euthanized before day 7, approximately 50% of lung tissue was affected. In the remaining animals the proportion of grossly affected lung tissue ranged from 3.5% to 50% of the total lung tissue.

The recovery of *App* from lung tissue by bacteriological examination and confirmatory PCR was successful in both animals euthanized prior to day 7 after challenge and in 16 of the remaining 22 challenged animals (73%) at day 21. The 6 bacteriologically negative pigs in the challenge group all had no gross lesions of *App* and all were classified as unaffected by CT score (Tables 1, 3).

Bacteriological examination of lung tissue of control animals resulted in the detection of *Bordetella bronchiseptica*, *E. coli* and alpha-haemolytic streptococci, while *App* was not isolated.

### Correlation of scores

On days 7 and 21, clinical scores significantly correlated with scores of both imaging techniques. On both days, radiographic scores positively correlated with computed tomography scores (r = 0.7757, P < 0.0001 and r = 0.5835, P = 0.0014, respectively). On day 21, the lung lesion scores were found to positively correlate with clinical scores, radiographic scores and with computed tomography scores. The Spearman’s rank correlation coefficient of the lung lesion scores with the CT scores (r = 0.8206, P < 0.0001) was higher compared to the radiographic scores (r = 0.4733, P = 0.0126). In order to translate the CT scores to four respiratory disease classes (not affected, slightly affected, moderately affected, severely affected), boundary values of the four CT score classes were chosen to achieve maximum concordance with the lung lesion score disease classes (Table 3c). An agreement in the assessment of disease severity with both methods was maximized by testing potential cut points for disease classes defined by the CT score in a contingency table. Disease classification by the different scoring schemes is summarized in Table 3.

On day 7, 55.2% of the animals were assigned to identical disease classes by both imaging methods, while 10.4% of the pigs were judged to be more affected based on x-ray and 34.4% were judged more severely ill by CT. On day 21, 55.6% of the animals were assigned to identical disease classes by CT and radiography, while 22.2% of the pigs were judged more severely ill by CT and 22.2% by x-ray compared to CT examination. Compared to gross pathology, results of radiographic examination at day 21 were accordance in 48.3% of pigs, while 17.2% were judged to be more affected according to radiography, i.e., 34.5% were judged as less affected by radiography. After maximization of the agreement between disease classes defined by the CT score and the established lung lesion score 82.8% of the pigs were assigned to identical disease classes by gross pathology and CT examination on day 21. 17.2% were classified as more severely affected by gross pathology than by CT examination, and no animals were assessed as more severely affected by CT. For the detection of lung changes, the positive predictive value of CT was calculated to be 100% and the negative predictive value was 83% (confidence limits 62–100%, 95% level of confidence).

## Discussion

The impact of computed tomography in veterinary medicine has increased during the last few years. Especially in small animals and horses CT is useful because of its routine feasibility and diagnostic reliability [23,24]. In contrast, CT has not been established as a routine imaging method in livestock medicine so far due to high investigation costs, time-consuming and personnel-intensive procedures and limited availability of equipment. In addition, CT examination of large animals is limited by the gantry diameter of most CT devices (600 mm) as well as size and capacity of the examination table with a maximum load of 160 kg [25]. Therefore, as described by Barbee and Allen (1990), CT examinations in horses are only carried out on the head, neck and limbs [25]. Computed tomography in pigs as animal models for human medicine has been used in several studies [26-28].

The aim of this study was to investigate the characteristic morphological alterations of lung tissue after experimental *App* challenge using computed tomography in pigs. Furthermore, the diagnostic potential and limitations of CT in pigs were compared with those of digital radiography. On the basis of the collected CT findings in living animals, a quantitative scoring system for determining the lung status of diseased pigs was derived. This scoring system is of high practical importance for follow-up studies during the course of experimental challenges with *App* but can not be transferred to other respiratory pathogens. The CT protocol used, based on 7 mm slices and a reconstruction interval of 5 mm was found appropriate for the detection of macroscopic lung lesions attributed to *App*. The detection of microscopic lung changes such as interstitial pneumonia (which may be a finding in viral pneumonias) is expected to require slices as narrow as 1 mm [29,30]. These conditions could not be achieved with the type of CT equipment and the short examination times used in our study. The examination time limit has not been determined in this study. It depends on the length of time the pigs remain anaesthetised and the speed of data capture by the CT equipment.

The clinical signs after experimental *App* challenge of 24 pigs ranged from nil to severe symptoms, with two affected animals requiring euthanasia due to severe dyspnoea on days 1 and 3, respectively. These results were reflected in the gross pathological findings which ranged from normal lungs to severe pleuropneumonia.

CT examination of animals in the challenged group showed ground-glass opacities and consolidations on days 7 and 21. Ground-glass opacities are caused by partial filling of the alveolar spaces, by thickening of the interstitium, partial collapse of alveoli, or an increased capillary blood volume [31,32]. As shown in several other studies, *App* infections are accompanied by swelling of capillary endothelia and thickening of alveolar septa in the first hours after challenge, followed by fibrinous exudation, migration of inflammatory cells and haemorrhage [16,17]. These pathological changes were found by CT examination in the form of hazy areas with an increase in lung density as well as more prominent bronchial and vascular outlines.

Consolidations are caused by an accumulation of transudate or exudate, which replace the alveolar air or by a collapse (atelectasis) in lobules. Therefore, they can be distinguished from ground-glass opacities because of the obscured underlying vessels and airway walls [32]. In the present study CT-diagnosed consolidations appeared as focal, homogeneous compaction of the lung with obliteration of the vascular and bronchial walls. These changes are due to focal necrosis, edematous and widened interstitia and serofibrinous exudates. According to the cross-sectional CT images, typical lesions caused by *App* were characteristically distributed throughout the lungs with a higher prevalence in the diaphragmatic lobes [5,8,10]. A quantitative measurement of lung tissue density for characterising macroscopic and microscopic lung tissues alterations using CT is possible, however, with some limitations [33,34]. The measurement of tissue density was used to characterise consolidations typical for *App* infection such as abscess-like nodules encapsulated in connective tissue. Galanski (1998) described the density value of abscesses ranging between 0 and 80 HU while pure pus has a CT value of 30 HU [33]. Others have found connective tissue formation and necrotic processes after experimental piglet infection with *Bordetella bronchiseptica* and *Pasteurella multocida* were of high densities (0 to 150 HU) in CT scans [35]. The average density of consolidations occurring in this study was also in the positive range of the Hounsfield scale compared to the physiological density of lung tissue which ranges from −550 HU to −950 HU [36]. Therefore, density measurement enabled a tentative diagnosis of the presence of accumulated pus, abscess-like nodules and necrotic areas.

Density measurements in areas with non-homogeneous, partially compacted and poorly defined ground-glass opacities cannot be performed [37]. Within these areas varying densities at the observation time occurred due to spontaneous breathing with varying inspiration depth. This variability resulted in high standard deviations of the measured values. Others have indicated that reproducible density measurements over time are only feasible at controlled inspiration depth [36].

Both the CT and the x-ray examinations strongly correlated with each other and with the clinical findings on days 7 and 21. However, for assessing the extent of lung alterations, a clinical scoring scheme alone is considered insufficient [38], due to the fact that severe tissue damage can be compensated by remaining parenchyma without clinical manifestation [1,21]. This observation is supported by our finding that clinical scores showed only a weak correlation with gross pathological findings at the end of the experiment.

Frequencies of cases within the severity classifications by CT, x-ray and necropsy were not consistent in all instances. As shown in this and other studies, pleurisy and pleural effusions could not be sufficiently differentiated from other gross pathological changes by CT and radiographic examination [21,39]. In some cases, false positive radiographic findings may occur especially in spontaneously breathing animals if the recordings are not carried out at the point of maximum inspiration [40-42]. False negative results may be due to the curvature of the diaphragm, hiding lung changes, including enlarged lymph nodes within the caudal lobes [43]. On the other hand, CT examination without overlapping organs, combined with cross-sectional images and a precise knowledge of anatomical structures enables the recognition of small lung tissue abnormalities. CT score graduation and its assignment to disease severity were chosen to ensure the best concordance with the disease classes defined by the lung lesion score. Therefore, the disease severity score graduation developed in the present study should be considered a preliminary guide for the quantification of *App* induced lung lesions and should be further validated in a larger cohort study. CT of the thorax could also be evaluated in caesarian-derived, colostrum-deprived or in gnotobiotic piglets, because these are widely used for challenge experiments in human medicine.

Our current approach compared the CT methodology with other methods that have been established in conventional pigs. Based on our findings, it is possible to detect gross pathological lung tissue changes in living pigs, which can be applied to efficacy studies of drugs and vaccines, or pathogenesis studies at certain points of time. In the context of ethical considerations, the number of animals can be reduced, since animals do not have to be sacrificed and necropsied at different time points to monitor the course of infection.

## Conclusion

CT thorax examination facilitated a high-contrast representation of characteristic but not pathognomonic *App* induced lung alterations. Although CT examination is superior to radiography and can therefore be recommended for scientific application, radiography can also be used for diagnosing gross pathological lung changes in subclinically-affected pigs. However, the additional assessment of disease history, histological examination and microbiological findings is still indispensable for accurate diagnosis of respiratory diseases in pigs.

## Methods

### Experimental setup

This study was conducted over a period of 21 days. After an acclimation period lasting several days, all animals were subjected to a daily general clinical examination. The animals were carefully monitored for signs of respiratory disease. After familiarisation, pigs in the challenge group were exposed to *App*, using an aerosol chamber. Control animals underwent mock challenge under the same conditions. On days 7 and 21, a digital radiographic and CT examination was performed. On day 21, all animals were euthanized, followed by necropsy and gross pathological examination of lung tissue.

### Animals

29 clinically healthy German Landrace pigs (male, neutered) aged 7 weeks, with an average body weight of 13.7 kg were used. Because it was known from previous experiments that approximately one third of the animals will not develop gross lung lesions, a total of 24 pigs were challenged. Pigs originated from a closed breeding herd of high health status that routinely tested seronegative for *App* and selected other pathogens on multiple occasions at six-monthly intervals. All pigs used in this study were tested negative for *App* antibodies prior to the study and were randomly allocated to a treatment group of 24 animals and a control group of 5 animals. The pigs were housed and cared for under the same conditions and in accordance with the Directive of the European Convention for the Protection of Vertebrate Animals Used for Experimental and Other Scientific Purposes (European Treaty Series, nos. 123 [http://conventions.coe.int/treaty/EN/treaties/html/123.htm] and 170 [http://conventions.coe.int/treaty/EN/treaties/html/170.htm]). Pigs of the control group were housed in an isolation unit separated from the group of challenged pigs. Air supply in each unit was assured by separated ventilation ducts. Challenged pigs and control pigs were looked after by different personnel to avoid cross-infection due to indirect contact between both groups. The study was approved from the local regulatory authorities and in accordance with the requirements of the national animal welfare law (approval number: 33.9-42502-04-05/919).

### Aerosol challenge

For aerosol challenge an *App* serovar 7 strain (AP76) was used, as this strain exhibits moderate virulence due to a lack of the cytotoxic and haemolytic Apx toxins I and III. Challenge experiments with strain AP76 have been performed on several occasions by our group and the dose chosen was previously shown to result in low mortality and chronic lung pathology [44,45].

*App* was cultured at 37°C and 5% CO_2_ in PPLO medium or on PPLO agar (Difco GmbH, Augsburg, Germany), supplemented with NAD (10 μg/ml; Merck, Darmstadt, Germany), L-cysteine hydrochloride (260 μg/ml; Sigma, Deisenhofen, Germany), L-cystine dihydrochloride (10 μg/ml; Sigma), dextrose (1 mg/ml; Roth, Karlsruhe, Germany), and Tween (0.1%). For preparation of challenge dose PPLO medium was inoculated with single colonies from PPLO agar and incubated overnight without shaking. Fresh PPLO medium was then inoculated with 10% of the overnight culture and incubated with shaking at 180 rpm to reach an OD of approximately 0.45 at 600 nm. This culture was subsequently diluted 1:15,000 with 0.9% sterile NaCl solution (154 mM). Animals were brought into an aerosol chamber in groups of four and 13 ml of the diluted culture (approximately 2 × 10^5^ colony forming units of *App*) was aerolized in the chamber within two minutes at a pressure of 2 bar [21,46-48]. Upon aerosolization a dose of approximately 1 × 10^2^*App* cells per litre of aerosol had been titrated in preliminary trials. Pigs were exposed for 10 mins to the aerosol in the closed chamber. An aliquot of the diluted culture was serially diluted and plated onto PPLO agar plates for overnight culture and retrospective confirmation of the challenge dose.

Animals in the control group underwent mock challenge under the same conditions using 0.9% sterile NaCl solution (154 mM).

### Clinical examination

Clinical examination was based on a clinical scoring system as previously described but without pulse oximetry monitoring [21]. Changes in general health as a result of a respiratory disease and clinically detectable deviations from the physiological breathing process were assessed. The scoring system included the following ten parameters: breathing noise, type of respiration, respiratory rate, coughing, colouration of the skin surface, posture, behaviour, feed intake, body temperature and vomiting. Cumulative clinical scores for each pig on day 7 and day 21 after challenge were calculated as the sum of scores for all daily examinations performed until that day (sum of daily clinical scores from day 1 to 7 and from day 1 to 21, respectively). Disease classifications by clinical scores on day 7 after challenge were: not affected (0–0.70), slightly affected (0.71–7.13), moderately affected (7.14–13.56), severely affected (>13.56). Disease classifications by clinical scores on day 21 after challenge were: not affected (0–2.00), slightly affected (2.01–34.70), moderately affected (34.71–67.3), severely affected (>67.3).

### Radiographic examination

For digital radiographic and computed tomographic examinations the animals were anaesthetised with Ketamine (15–20 mg/kg body weight intramusculary (i.m.), Ursotamin, Serum-Werk Bernburg-AG, Bernburg, Germany) and Azaperone (2 mg/kg body weight i.m., Stresnil, Janssen-Cilag GmbH, Baar, Switzerland). The radiographic and CT results were judged by one investigator.

### Digital x-ray examination

Digital x-ray examination of the thorax was performed with a 80 kV no-scatter grid and automatic exposure (Precimat, manufactured by Picker International., Munich, Germany). To assess the left and right lung, recordings were made using a lateral path of x-rays on both sides. For this purpose the animals were placed in lateral recumbency. A dorso-ventral recording was then performed in sternal recumbency with the legs extended [49]. The film-focus distance was 1.5 m. Image plate cassettes (EURAS MedTech, PROVOTEC, X-RAY, Espelkamp, Hannover, Germany) sized 240 × 300 mm were used and readout linearly with the iCR 3600, a digital non-contact image storage system (EURAS MedTech, PROVOTEC, X-RAY, Espelkamp, Hannover, Germany). A class A black and white monitor (firm, flat Medic) with the software digipaX (digipaX GmbH, Leipzig, Germany) was used to view the images, which were interpreted objectively following a previously published radiographic score system [21] based on dividing the right and left lung into quadrants (eight sections). Each quadrant was evaluated by examination of the dorso-ventral and lateral views. Scores for various radiographic findings (Table 2) were added to determine the score of the respective quadrant. Finally, the added scores of the eight quadrants provided the radiographic score for the whole lung.

### CT examination

A third generation single-slice spiral CT (Tomoscan M, Philips Company, Hamburg, Germany) was used for the investigations. The scanogram and volume scan was performed with the following settings: tube voltage 120 kV, current 40 mA, slice thickness 7 mm, reconstruction interval 5 mm, pitch 1.5. The matrix was 512 × 512 pixels. The animals were placed symmetrically in sternal recumbency and scanned from the cranial thoracic aperture to the caudal end of the lung. Imaging was performed without a contrast agent. A standard Windows PC and an image reporting and processing software for medical diagnostics (e-film, eFilm Medical Inc., Toronto, Ontario, Canada) were used to save and evaluate the absorption and density measurements of the CT scans. The CT images were displayed with conventional window settings specific for lung tissue (window width, 1500 Hounsfield units (HU); and window level - 600 HU). CT images were assessed visually by one blinded investigator and pathological changes were evaluated for morphology (ground glass opacity, consolidation), distribution pattern, localisation and absorption density.

In order to describe the localisation of lesions, the lung area was divided into three sections, i.e. cranial, middle, caudal. Because lung lobes are not vertically defined they overlap multiple vertebrae so that the compartmentation serves only for rough orientation: The cranial section was located between the first and the fourth thoracic vertebrae and contained mainly the apical lobes. The middle section was located between the fourth and the sixth thoracic vertebrae and contained mainly the cardiac lobes. Finally, the caudal section was located between the sixth and the eleventh vertebrae and contained predominantly the diaphragmatic lobes and the accessory lobe. For each transverse cross-section of the lung, four quadrants (left-dorsal, left-ventral, right-dorsal, right-ventral), were examined for pneumonic changes [50]. Consolidations and ground glass opacities were scored, with one scoring point being assigned for each involved quadrant. Pathological changes in all four quadrants resulted in a maximum score of four points.

The CT scoring system also included density assessment of the consolidations detected. Absorption density measurement was based on selecting the centre of a consolidation in any transverse section as a circular region of interest (ROI) with an area of 120 mm^2^ as previously described [51]. The mean density value (HU; mean Hounsfield Unit) and standard deviation were calculated from the absorption density of the pixels in the selected area. When scoring each transverse cross section, the consolidation with the highest density was taken into account as follows: < − 550 HU no scoring points; from - 550 to - 150 HU two scoring points; and > − 150 HU four scoring points. Thus the scores for the number of pathologically altered quadrants per image (maximum of four) and the scores for consolidations based on the highest density (maximum of four) were added and divided by the total number of transverse sectional images, so that a maximum of eight points per pig could be achieved.

Spearman’s rank correlation coefficients between the individual scores (clinical, radiographical, CT, lung lesion) were calculated for days 7 and 21. Pigs were allocated to ranges of CT scans based on findings of gross pathology (lung lesion scores), which indicated CT cutpoints of 0.24, 1.71 and 2.23 gave the best correlation with slight, moderate and severe disease respectively.

### Pathological and bacteriological examination

At necropsy, lungs were examined by gross pathology. Findings were quantified using the Lung Lesion Score (LLS) proposed by Hannan et al. (1982) and specified in the European Pharmacopoeia for vaccine development [22,52]. For this purpose, a schematic map of the porcine lung was used. In this map the lung was subdivided into 74 triangles, with 7 in each of the apical and cardiac lobes, 8 in the accessory lobe and 19 in each diaphragmatic lobe. Each lobe may contribute a maximum score of 5. To determine the LLS, the damaged area was determined by counting the number of triangles with pathological changes and expressing it as a fraction of five for each lobe. The values for each lung lobe were summed, resulting in an LLS with a maximum possible score of 35.

Reisolation of *App* was achieved by plating tissue of each of the seven lung lobes on selective meat and blood agar [53]. Bacteriological confirmation of *App* was subsequently performed by PCR for the ApxIIA gene and by testing for urease activity [44,47,54]. Lung tissue of challenged animals was not examined for other bacterial species, while lung tissue of animals from the control group was examined for *App* and other bacterial species.

## Authors’ contributions

CB carried out the clinical studies, participated in the statistical analysis and developed the CT score and drafted the manuscript. IHP was involved in clinical examinations and necropsies and helped to draft the manuscript. DH and FFRB organized all clinical studies and carried out the aerosol challenge. MB participated in the statistical analysis and helped to develop the CT score. HG participated in necropsies and prepared and stained histology sections. GFG performed the aerosol challenge and interpreted the serological investigation. KHW participated in the design and the coordination of the study. All authors read and approved the final manuscript.
